# Associations of Infant Feeding and Timing of Weight Gain and Linear Growth during Early Life with Childhood Blood Pressure: Findings from a Prospective Population Based Cohort Study

**DOI:** 10.1371/journal.pone.0166281

**Published:** 2016-11-10

**Authors:** Marieke de Beer, Tanja G. M. Vrijkotte, Caroline H. D. Fall, Manon van Eijsden, Clive Osmond, Reinoud J. B. J. Gemke

**Affiliations:** 1 Department of Pediatrics, VU University Medical Center, Amsterdam, The Netherlands; 2 Department of Social Medicine, Academic Medical Center, University of Amsterdam, Amsterdam, The Netherlands; 3 MRC Lifecourse Epidemiology Unit, Southampton General Hospital, University of Southampton, Southampton, United Kingdom; 4 Department of Epidemiology, Documentation and Health Promotion, Public Health Service, Amsterdam, The Netherlands; Centro Cardiologico Monzino, ITALY

## Abstract

**Objective:**

Small birth size and rapid postnatal growth have been associated with higher future blood pressure. The timing of these effects, the relative importance of weight gain and linear growth and the role of infant feeding need to be clarified.

**Methods:**

We assessed how blood pressure relates to birth weight, infant and childhood growth and infant feeding (duration of exclusive breastfeeding and timing of introduction of complementary feeding) in 2227 children aged 5 years from a prospective cohort study (Amsterdam Born Children and their Development). Postnatal growth was represented by statistically independent measures of relative weight gain (weight gain independent of height) and linear growth in four age periods during infancy (0–1 month; 1–3 months; 3–6 months; 6–12 months) and from 12 months to 5 years.

**Results:**

Lower birth weight was associated with higher childhood diastolic blood pressure (-0.38 mm Hg.SD^-1^; P = 0.007). Faster relative weight gain and linear growth after 1 month were positively associated with systolic and diastolic blood pressure. Associations of linear growth with systolic blood pressure ranged from 0.47 to 1.49 mm Hg.SD^-1^; P<0.01 for all. Coefficients were similar for different periods of infancy and also for relative weight gain and linear growth. Compared to breastfeeding <1 month, breastfeeding >1 month was associated with lower blood pressure (e.g. >6 months -1.56 mm Hg systolic blood pressure; P<0.001). Compared to >6 months, introduction of complementary feeding <6 months was associated with higher blood pressure (e.g. 4–6 months 0.91 mm Hg systolic blood pressure; P = 0.004).

**Conclusions:**

After the age of one month faster growth in either weight or height is associated with higher childhood blood pressure. It is unknown whether faster weight gain and linear growth carry the same risk for adult hypertension and cardiovascular morbidity. Longer breastfeeding and delayed introduction of complementary feeding may be associated with lower adult blood pressure.

## Introduction

The World Health Organization has attributed 13% of deaths worldwide to raised blood pressure (BP), making it one of the most important modifiable cardiovascular risk factors globally.[1] Data from diverse populations have shown that blood pressure tracks from childhood to adulthood, with average correlation coefficients around 0.3–0.4.[2] Fetal growth restriction has been identified as a risk factor for raised BP in later life.[3,4] Studies addressing the relationship of weight gain in infancy to later life BP have shown mixed results. The Helsinki Birth Cohort study showed an inverse association between infant weight gain and later life BP.[5] Some studies, including the Hertfordshire, Brompton, Hong Kong and Northern Finland Birth cohorts, showed no association.[6–9] The Barry Caerphilly Growth, COMPASS, Pelotas birth cohort and ALSPAC studies showed a positive association between infant weight gain and later life BP.[10–13] Two of these studies suggested that it is weight gain in early infancy (the first 5–6 months of life) that is most important.[10,12] There is therefore confusion about what constitutes optimal infant weight gain for future cardiovascular health.

Greater weight gain in childhood (*after* infancy) has been associated with higher later life BP in numerous studies.[5,8,12–14] It is not clear, however, whether the relationship of weight gain to later life BP comes from the component of weight gain that is due to linear (skeletal) growth or to soft tissue (lean and fat) growth. Knowing this may provide insight into the mechanisms linking postnatal growth and later life BP. The Amsterdam Born Children and their Development (ABCD) study has measurements of both weight and height, and was unusual in collecting these at frequent intervals in infancy.

In the ABCD study we found that longer duration of breastfeeding and later introduction of complementary feeding was associated with slower growth in infancy[15] which was in accordance with other studies.[16,17] Early feeding may therefore be related to later life BP. Furthermore, infant feeding may have direct programming effects on later life BP, for example due to differences in sodium intake[18,19], long-chain polyunsaturated fatty acids (LCPUFAs)[19,20] or epigenetic effects.[21] Breastfed compared with formula-fed infants have been shown to have lower adult BP in some[22,23] but not all studies.[24–26]

The role of complementary feeding has received little attention until now. In two cohort studies, no association was found between timing of introduction of complementary feeding and childhood BP.[24,27] However, recent findings from the Generation R study group showed that later introduction of complementary feeding was associated with lower childhood BP.[28]

The aims of our study were to answer the following questions in a population based cohort 1) Does growth in either weight or height during infancy have significant positive or negative associations with BP at age 5 years?; 2) If so, are there ages and periods in infancy when the associations are stronger?; 3) Is the association of childhood weight gain with BP related to linear growth or soft tissue growth?; 4) Are duration of breastfeeding and timing of introduction of complementary feeding associated with BP at 5 years?; 5) If so, does growth in infancy have a mediating role?

## Methods

The present study is part of the Amsterdam Born Children and their Development (ABCD) study, a population based prospective, longitudinal pregnancy cohort.[29]

### Study population

**Fig 1:**
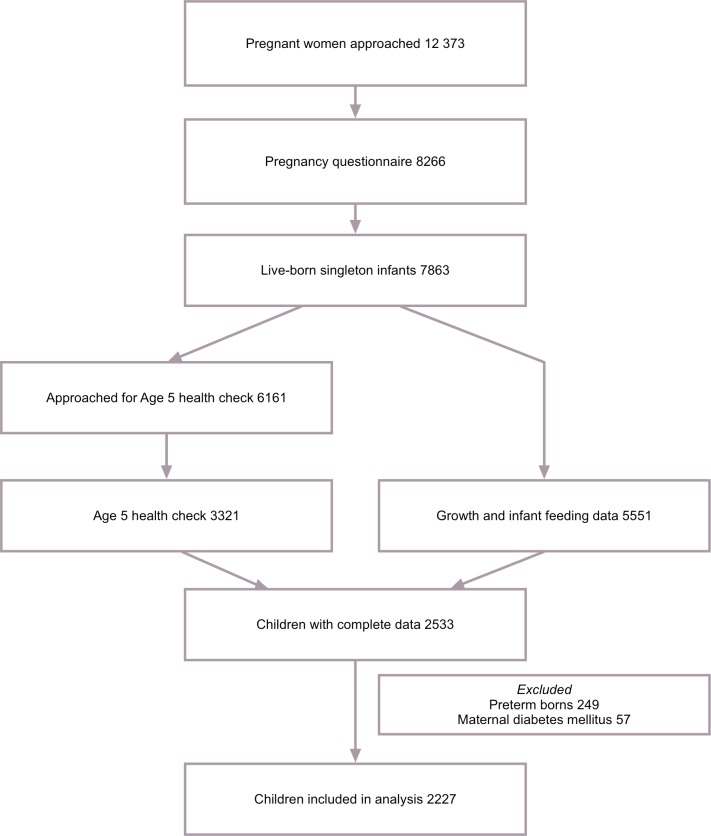
Sampling procedure.

Between January 2003 and March 2004, all pregnant women living in Amsterdam were invited to participate in this study at their first visit (12–14th week of pregnancy) to an obstetric caregiver (Fig 1). A questionnaire, including sociodemographic data, obstetric history and lifestyle, was sent to the pregnant woman's home address. Questionnaires were returned by 8266 women (response rate 67%). From this group 7863 gave birth to a viable singleton infant. Pregnancy duration, gender and birth weight were obtained from Youth Health Care (YHC) centers, which perform neonatal screening for congenital inborn errors of metabolism in all Dutch newborns. The YHC centers routinely invited them from birth onwards for regular health evaluations at set ages: 8 times between birth and 12 months of age: around 1, 2, 3, 4, 6, 7.5, 9 and 11 months. During these check-ups weight and supine height were measured and feeding patterns (duration of breastfeeding and timing of introduction of complementary feeding) were monitored. When measuring height the parent was asked to hold the infant’s head in contact with a fixed board, and the YHC health worker stretched the infant out to its maximum length and then brought a moving board into contact with the heels. Growth- and infant feeding data were collected for a total of 5551 children. When the children reached the age of 5 years, the addresses of 6161 mothers were retrieved from the YHC registry. The mothers received an informed consent sheet for a health check of their child, for which 4158 (67%) women gave permission. The health check included measurements by trained research assistants, including anthropometry and BP, and was carried out in 3321 children aged 5 years (2008–2010).[30] The current study population included children whose birth weight and growth and infant feeding data were available, as well as anthropometry and BP at age 5 years (n = 2533). Babies born preterm (pregnancy duration less than 37 weeks, n = 249) and babies of mothers with pre-existing or gestational diabetes mellitus (n = 57) were excluded, leaving 2227 children for analysis.

### Exposure variables: birth weight, postnatal growth and infant feeding

We used birth weight (birth height is not routinely measured in the Netherlands) and subsequent weight and height at 1, 3, 6, and 12 months and 5 years of age. Using the maximum number of measurements available at all time points, sex-specific internal z scores were generated. If a measurement was not recorded at the exact age we derived this value by interpolating between the nearest measurements. Because children without birth weight or 5-year measurements were excluded we did not need to extrapolate these measures. The age ranges accepted for interpolation at 1, 3, 6 and 12 months were 0–2 months, 2–4 months, 4–8 months and 9–15 months respectively. 95% of measurements were made within 0.3, 0.3, 0.4, and 0.9 months respectively from the exact age.

Using these measures of size, we derived measures of growth (i.e. change in size). Disentangling the consequences of weight gain and linear growth at different ages requires statistical methods to address the high correlation of weight with height, and of repeated measurements in the same individual over time. Our “conditional” size measures were standardized residuals derived from regressing current size on all prior size measures.[7,12–14,31–33] Conditional relative weight was current weight accounting for current height and all prior weight and height measures. Conditional height was current height accounting for prior height and weight measures (but not current weight). Current weight was not accounted for in constructing conditional height because it does not contribute to current height. In contrast we accounted for current height in constructing conditional weight because it does contribute to current weight. For example, childhood conditional relative weight was derived from regressing 5-year weight on 5-year height, weights and heights at 1, 3, 6 and 12 months, and birth weight. Conditional variables represent children’s deviation from expected size, based on their own previous size and the growth of the other children in the study population, and can be interpreted as representing faster or slower linear growth or relative weight gain. Conditional regression analysis is designed to assess growth over distinct age periods and to eliminate collinearity problems caused by repeated measures.[33] Although the age intervals chosen increased in length, the correlations between measurements at the beginning and end of each interval were similar, as the child’s growth stabilised. This justified the selection of shorter intervals at younger ages.

Duration of exclusive breastfeeding (excluding any other form of nutrition including formula feeding and complementary feeding) was categorized into 4 subgroups: no exclusive breastfeeding or < 1 month, 1–3 months, 3–6 months and > 6 months. Timing of introduction of complementary feeding (defined as any food other than breastfeeding or formula feeding) was divided into 3 subgroups: < 4 months, between 4–6 months and > 6 months.

### Outcome measures

Outcome measures were systolic BP and diastolic BP at age 5 years. BP in the right arm was measured three times after five minutes rest in the supine position using an Omron 705 IT (Omron Healthcare Inc., Bannockburn, IL, USA) with an age appropriate cuff. The first measurement was discarded and the last two measurements were averaged for the analysis.

### Confounding variables

Potential confounding variables that we considered were: the child’s sex (male, female) and exact age (continuous, years) at the 5-year health check, and the following maternal characteristics: age (continuous, years), pre-pregnancy body mass index (BMI, continuous, kg/m^2^), height (continuous, cm), educational level (years of education after primary school, continuous), primiparity (yes, no), use of alcohol during pregnancy (yes, no), smoking during pregnancy (no, 1–5 cigarettes / day, ≥ 6 cigarettes / day), hypertension (none, pre-existing hypertension, pregnancy-induced hypertension), ethnicity (based on mother’s country of birth: Dutch, Surinamese, Turkish, Moroccan, other) and pregnancy duration (continuous, weeks).[30]

### Statistical analyses

We used multivariable linear regression to estimate associations of birth weight, conditional relative weight and conditional height 0–1, 1–3, 3–6, 6–12 months (infancy) and 12 months-5 years (childhood) with BP at age 5 years, adjusting for all the above-mentioned potential confounding variables. We examined the association of infant feeding with BP using the same approach, further adjusting for birth weight. When looking at timing of introduction of complementary feeding as the exposure variable we adjusted for duration of full breastfeeding (defined as no formula feeding). To examine the potential mediating role of growth in infancy in the relation of infant feeding and childhood BP, we further adjusted for conditional relative weight and conditional height in infancy (all variables representing conditional relative weight and conditional height 0–1, 1–3, 3–6 and 6–12 months were additionally included in the model). Both exclusive breastfeeding and timing of introduction of complementary feeding were treated as categorical variables. Statistical analysis was carried out using IBM SPSS for Mac (version 20.0.0).

### Ethics committee approval

Approval was obtained from the VU University Medical Center Medical Ethical Committee, Academic Medical Center Medical Ethical Committee, and the Registration Committee of Amsterdam. All participants gave written informed consent for themselves and their children.

## Results

### Descriptives

Compared to the cohort members who were not studied (n = 3324), the study group (n = 2227) mothers were older (mean 32.2 vs. 30.2 years, P<0.001), had a lower pre-pregnancy BMI (22.9 vs. 23.2 kg/m^2^, P = 0.003) and taller stature (mean 1.70 vs. 1.68 m, P<0.001). They were less likely to smoke during pregnancy (5.8 vs. 7.3%, P = 0.015), more likely to drink alcohol during pregnancy (28.3 vs. 18.8%, P<0.001), more likely to be of Dutch origin (43.2 vs. 22.1%, P<0.001), less likely to have pre-existing hypertension (2.4 vs. 4.5%, P<0.001), had a longer pregnancy duration (40.1 vs. 39.6 weeks, P<0.001), were more likely to initiate exclusive breastfeeding (77 vs. 71%, P<0.001) and tended to breastfeed for longer (e.g. 18.7 vs. 15.2%, P = 0.001 continued for > 6 months). The offspring had a higher birth weight (mean z score 0.08 vs. -0.06, P<0.001) and slower relative weight gain between 1–3 months (-0.05 vs. 0.05, P = 0.001) and 3–6 months (-0.03 vs. 0.03, P = 0.04). They had faster linear growth between 0–1 month (mean z scores 0.03 vs. -0.03, P = 0.039) and slower linear growth between 1–3 months (-0.04 vs. 0.03, P = 0.017) and 3–6 months (-0.06 vs. 0.05, P<0.001). These differences between responders and non-responders were similar for people of Dutch and non-Dutch origin. The characteristics of the study sample are shown in Table 1. Using the WHO growth standards (www.who.int) mean weights and heights at the different time points in the first year varied between 0 and 0.5 SD. Mean weight and height at 5 years were around 1 and 1.5 SD respectively (S1 Table).

**Table 1:** Maternal and child characteristics by sex (N = 2227).

Measurement	Boys (n = 1146)		Girls (n = 1081)	
	Mean	SD	Mean	SD
**Mother**				
Age (y)	32.2	4.4	32.2	4.3
Pre-pregnancy BMI (kg/m^2^)	22.9	3.7	22.9	3.6
Height (m)	1.70	0.07	1.69	0.07
Education after primary school (y)	10.0	3.7	10.0	3.5
Primiparous (% yes)	55.3		54.4	
Alcohol (% yes)	28.4		28.1	
*Smoking*				
Non-smoking (%)	94.2		94.2	
1–5 cigarettes/day (%)	3.6		3.1	
≥ 6 cigarettes/day (%)	2.3		2.8	
*Ethnicity*				
Dutch (%)	78.0		77.7	
Surinamese (%)	2.8		2.0	
Turkish (%)	2.1		1.9	
Moroccan (%)	5.4		3.3	
Other (%)	11.7		15.0	
*Hypertension*				
Pre-existing hypertension (%)	2.4		2.4	
Gestational hypertension (%)	8.1		9.1	
**Child–At birth**				
Pregnancy duration (weeks)	40.2	1.2	40.1	1.2
Birth weight (kg)	3.61	0.50	3.47	0.46
Small for gestational age (<P10)[34](%)	8.9		7.9	
Large for gestational age (>P90)[34](%)	11.1		10.4	
**Child– 1–12 months measurements**				
Weight (kg)				
1 month	4.57	0.54	4.28	0.49
3 months	6.40	0.68	5.83	0.62
6 months	8.09	0.82	7.45	0.76
12 months	10.21	1.01	9.53	0.99
Height (cm)				
1 month	55.2	2.0	54.1	2.0
3 months	62.0	2.0	60.4	1.9
6 months	68.4	2.1	66.6	2.0
12 months	76.5	2.4	74.9	2.3
*Duration of exclusive breastfeeding (months)*				
< 1 month (%)	53.1		53.4	
1–3 months (%)	13.6		13.0	
3–6 months (%)	18.4		20.2	
> 6 months (%)	14.9		13.4	
*Age at introduction of complementary feeding (months)*				
< 4 months (%)	5.9		5.2	
4–6 months (%)	37.6		37.2	
> 6 months (%)	56.5		57.6	
**Child–At age 5 health check**				
Age (y)	5.8	0.5	5.8	0.5
Weight (kg)	21.2	3.0	21.0	3.5
Height (cm)	116.9	5.6	116.2	5.7
BMI (kg/m^2^)	15.5	1.3	15.5	1.6
Systolic BP (mm Hg)	99.0	7.0	99.0	7.1
Diastolic BP (mm Hg)	56.0	5.7	57.8	6.0

BP − blood pressure; P10 - 10^th^ percentile of the Dutch reference [34]; P90 – 90^th^ percentile of the Dutch reference [34]

### Confounding variables

The associations of confounding variables with size and growth (S2 Table), BP (S3 Table) and infant feeding (S4 and S5 Tables) are presented as supplementary material. To summarize these data briefly, lower birth weight was associated with the following maternal characteristics: younger age, lower pre-pregnancy BMI, shorter stature, lower educational level, smoking during pregnancy, non-use of alcohol, primiparity, non-Dutch ethnicity, gestational hypertension and shorter pregnancy duration. Faster relative weight gain and linear growth tended to be associated with younger maternal age, higher pre-pregnancy BMI, lower educational level, smoking during pregnancy, non-use of alcohol and pre-existing hypertension. Faster linear growth was additionally associated with taller maternal stature and shorter pregnancy duration. Exceptions to the above were that faster linear growth during 0–1 months was associated with lower maternal BMI, higher educational level, not smoking during pregnancy and longer pregnancy duration. There were significant differences in growth between ethnic groups (S2 Table).

Younger maternal age, higher pre-pregnancy BMI, lower educational level, pre-existing hypertension, smoking during pregnancy and non-use of alcohol were associated with higher childhood BP in the offspring (S3 Table).

Women who exclusively breastfed for 3–6 months were older (P = 0.05), thinner (P = 0.04), better-educated (P<0.001), and had longer pregnancies (P = 0.001), than women who breastfed for <1 month. They were less likely to smoke (P = 0.02 and 0.01), have pre-existing hypertension during pregnancy (P = 0.03) and be of Surinamese (P = 0.01) or Moroccan (P = 0.01) origin, than women who breastfed for less than one month (S4 Table). Findings for women who breastfed for > 6 months were similar regarding age, educational level and smoking. In addition these women were less often primiparous, consumed less alcohol during pregnancy and were more likely to be of other origin than women who breastfed for <1 month (S4 Table).

Women who introduced complementary feeding >6 months were older (P = 0.01), thinner (P = 0.01) and better-educated (P<0.001). They were less likely to smoke (P = 0.003–0.05), to have hypertension during pregnancy (P = 0.01) and to be of Surinamese origin (P = 0.003) than women who introduced complementary feeding <6 months (S5 Table).

### Relationships of birth weight, relative weight gain and linear growth in infancy and childhood with childhood blood pressure

Lower birth weight was associated with higher childhood diastolic BP (Table 2). Being small for gestational age or large for gestational age did not modify this association (not shown). Faster relative weight gain after the first month (i.e. 1–3, 3–6 6–12 months and 12 months-5 years) was associated with higher systolic and diastolic BP in childhood (Table 2). Similar associations were found for linear growth after 1–3 months. The strength of the associations was comparable for the different age intervals in infancy, while the associations of growth from 12 months-5 years with BP were 2–3 times stronger than those of infant growth. Associations for relative weight gain were comparable to those for linear growth. Having a BMI > 2 SD at age 5 years according to WHO growth standards (www.who.int) did not modify the associations found (not shown).

**Table 2:** Relationships of birth weight, relative weight gain and linear growth (all expressed in z scores) with childhood systolic blood pressure (mm Hg) and diastolic blood pressure (mm Hg).

	Systolic BP (mm Hg)			Diastolic BP (mm Hg)		
	B	95% CI		B	95% CI	
Birth weight	-0.18	-0.50	0.14	-0.38	-0.66	-0.11
Relative weight gain 0–1 m	0.09	-0.19	0.36	0.03	-0.21	0.26
Relative weight gain 1–3 m	0.73	0.45	1.01	0.27	0.02	0.51
Relative weight gain 3–6 m	0.86	0.59	1.14	0.46	0.23	0.70
Relative weight gain 6–12 m	0.71	0.44	0.98	0.26	0.02	0.49
Relative weight gain 12 m-5 y	1.36	1.08	1.63	0.92	0.68	1.16
Linear growth 0–1 m	0.20	-0.08	0.49	0.00	-0.25	0.24
Linear growth 1–3 m	0.47	0.20	0.75	0.11	-0.13	0.35
Linear growth 3–6 m	0.65	0.37	0.93	0.34	0.10	0.59
Linear growth 6–12 m	0.53	0.25	0.80	0.29	0.05	0.53
Linear growth 12 m-5 y	1.49	1.21	1.77	0.51	0.27	0.76

B values are linear regression coefficients indicating the change in BP (in mm Hg) per standard deviation change in the exposure. The analyses were adjusted for the child’s age and sex and maternal age, pre-pregnancy BMI, height, educational level, primiparity, ethnicity, smoking, use of alcohol, hypertension and pregnancy duration. BP−blood pressure

### Relationship of feeding type with childhood blood pressure

Compared with breastfeeding for <1 month, exclusive breastfeeding for >1 month and for 3–6 months were associated with lower systolic and diastolic BP respectively (Table 3).

**Table 3:** Relationships of duration of exclusive breastfeeding and timing of introduction of complementary feeding with childhood blood pressure (mm Hg).

	Systolic BP (mm Hg)							Diastolic BP (mm Hg)						
		Model 1			Model 2				Model 1			Model 2		
	(mean, SD)	B	95% CI		B	95% CI		(mean, SD)	B	95% CI		B	95% CI	
**Duration of exclusive breast feeding**														
<1 m (n = 1185) ref	(99.7, 7.4)	-	-	-	-	-	-	(57.3, 6.0)	-	-	-	-	-	-
1–3 m (n = 297)	(98.5, 6.6)	-1.18	-2.06	-0.30	-1.03	-1.89	-0.17	(56.9, 6.3)	-0.33	-1.06	0.41	-0.27	-1.00	0.46
3–6 m (n = 429)	(98.3, 6.5)	-1.02	-1.80	-0.25	-0.60	-1.38	0.17	(56.0, 5.4)	-1.02	-1.67	-0.38	-0.83	-1.48	-0.18
>6 m (n = 316)	(97.9, 6.5)	-1.56	-2.43	-0.69	-0.77	-1.65	0.12	(56.7, 5.7)	-0.48	-1.20	0.25	-0.10	-0.84	0.65
**Timing of introduction of complementary feeding**														
<4 m (n = 117)	(100.4, 7.5)	1.16	-0.19	2.50	0.76	-0.56	2.07	(58.2, 5.5)	1.21	0.10	2.33	1.04	-0.08	2.15
4–6 m (n = 826)	(99.6, 7.3)	0.91	0.29	1.53	0.72	0.11	1.33	(57.2, 6.1)	0.47	-0.05	0.98	0.38	-0.14	0.89
>6 m (n = 1260) ref	(98.5, 6.8)	-	-	-	-	-	-	(56.6, 5.8)	-	-	-	-	-	-

B values are linear regression coefficients indicating the change in BP (in mm Hg) for each category of the feeding variables compared with the reference category. (Model 1) Adjusting for the child’s birth weight, age and sex and maternal age, pre-pregnancy BMI, height, educational level, primiparity, ethnicity, smoking, use of alcohol, hypertension and pregnancy duration; (Model 2) as in model 1 but with the addition of relative weight gain and linear growth for all age periods of infancy. BP−blood pressure.

Compared with the introduction of complementary feeding >6 months, their introduction <4 months tended to be associated with higher systolic (P = 0.09) and diastolic BP (P = 0.03). Introduction of complementary feeding between 4–6 months was associated with higher systolic (P = 0.004) and diastolic (P = 0.08) BP (Table 3). Adjusting for growth in infancy attenuated these associations, indicating a possible mediating role of growth in infancy. Having a BMI > 2 SD at age 5 years according to WHO growth standards (www.who.int) did not modify the associations found (not shown).

## Discussion

This study showed that faster relative weight gain and faster linear growth after 1–3 postnatal months were associated with higher systolic and diastolic BP at age 5 years. In contrast, growth during the first postnatal month was unrelated to BP. Coefficients were similar for growth during the different periods of infancy (after 1–3 months), and were stronger for growth between 1 and 5 years. They were similar for relative weight gain and linear growth. Exclusive breastfeeding for longer than 1 month and introduction of complementary feeding after 6 months were associated with lower childhood BP, and these associations appeared to be partly mediated through growth in infancy.

Conditional relative weight gain and linear growth are, by construction, uncorrelated, enabling us to separate the associations of BP with relative weight gain from those with linear growth. By expressing them in SD units, we were able to compare the size of their effects, and compare effects at different ages. Weight gain is a result of both soft tissue gain and linear growth; our relative weight gain variables represent soft tissue growth distinct from skeletal growth and therefore give more information than weight gain alone, which is correlated with height gain.

A 1 SD increase in relative weight in infancy was associated with a 0.71 to 0.86 mm Hg higher childhood systolic BP. This is higher than the associations found in the UK ALSPAC study (+1 SD increase in weight-for-height in infancy was associated with a 0.25 to 0.4 mm Hg higher adolescent systolic BP)[13] and lower than those found in the UK Barry Caerphilly Growth study, the COMPASS study in Sweden and the Pelotas birth cohort study in Brazil (+1 SD increase in weight in infancy was associated with a 1 to 1.79 mm Hg higher adolescent/adult systolic BP).[10–12] The associations of infant growth with childhood diastolic BP in our study were also weaker than associations found in the Barry Caerphilly study (0.26 to 0.46 mm Hg. SD^-1^ vs 0.74 mm Hg. SD^-1^).[10] The other cohort studies either did not find associations with diastolic BP[13] or did not have diastolic BP as an outcome measure.[11,12] The Barry Caerphilly study and Pelotas birth cohort study suggested that weight gain in *early* infancy (5–6 months) is most important[10,12], however they did not look at separate age intervals within this period. We found that relative weight gain and linear growth in the first month were unrelated to childhood BP. Only one other study that we are aware of looked at separate age intervals in the first postnatal months.[13] Similar to our findings, this study showed that relative weight gain between 2.9 weeks and 1.6 months, and linear growth in the first 2 months were unrelated to adolescent BP.[13] We are unable to explain the mechanism(s) by which growth in the first postnatal month may have different associations with later BP.

Associations of childhood relative weight gain and linear growth with childhood BP were equal in strength, a finding that is consistent with 3 other large cohort studies.[5,13,14] In contrast, in the Pelotas birth cohort study, childhood weight gain, but not linear growth was associated with adolescent BP.[12] Another large birth cohort study in Hong Kong showed that higher BMI gain between 3–7 years and greater linear growth between 7–11 years predicted higher adolescent BP.[8] The positive association between relative weight gain and childhood BP may be related to the same mechanisms which are thought to cause adiposity-related higher BP, namely activation of the sympathetic nervous system, primary sodium retention, increased renin activity, increased levels of angiotensinogen and aldosterone, insulin resistance and inflammation.[35] However, relative weight gain comprises both fat and lean tissue gain. Gain in lean mass is regulated by insulin-like growth factor-1 during infancy, and by growth hormone after infancy[36], these hormones have been associated with changes in blood vessel structure and later higher BP.[26] We were not able, in our study, to distinguish between fat and lean tissue gain in our infants and children. It has been suggested that the positive relationship between height and systolic BP may represent a physiological adaptation by which the cardiovascular system accommodates to the relative length of the arterial tree by a commensurate variation in perfusion pressure.[37,38] It is not clear whether this has pathological consequences, but the evidence suggests not; taller people actually have a lower risk of cardiovascular disease, despite higher BP.[39,40]

Compared with exclusive breastfeeding for less than 1 month, exclusive breastfeeding for longer than 3 months was associated with a 1.02 to 1.56 mm Hg lower childhood systolic BP. The associations appeared to be partly mediated through growth in infancy, however no formal tests were performed to prove mediation. This is consistent with findings from a meta-analysis in 2005, which showed that initiation of breastfeeding was associated with a 0.6 mm Hg lower later life systolic BP.[23] A follow-up study at age 13–16 years of preterm infants, randomized to breast milk or preterm formula showed that the proportion of human milk in the neonatal period was inversely related to later mean arterial BP.[22] However, the findings differ from those of the PROBIT study, a large randomized controlled trial (RCT) of breastfeeding promotion.[41] There were no differences in systolic or diastolic blood pressure between children in the intervention and control groups at 6.5 years.[41] RCTs in general yield a higher level of evidence than observational studies, but the difference in duration of breastfeeding between the intervention and control groups in PROBIT (72.7% vs. 60% of mothers were still breastfeeding at 3 months) may have been too small to produce a detectable difference in BP.[41]

The introduction of complementary feeding before 4 months and between 4–6 months was associated with a 1.21 mm Hg higher diastolic and a 0.91 mm Hg higher systolic BP respectively when compared with the introduction of complementary feeding after 6 months. This is consistent with recent findings from the Generation R study group showing that introduction of complementary feeding before compared with after 5 months was associated with a 0.86 mm Hg higher diastolic and a 0.94 mm Hg higher systolic BP.[28] In our study, the association appeared to be partly mediated through growth in infancy, however no formal tests were performed to prove mediation. Higher sodium intake due to earlier introduction of complementary feeding may be another factor.[18,19] Although an association with childhood overweight may partially explain our findings[15], controlling for 5-year BMI and height hardly altered the effect estimates (our data; not shown).

### Strengths and limitations

Strengths of our study were that it was a large community based cohort study with extensive prospective data on confounding factors. In contrast to some other large studies, anthropometry was carried out by professional health workers, as was the collection of information about infant feeding, and childhood BP was measured by trained research assistants. Measurements were made in the Youth Health Care centers at slightly varying ages, but always within days or weeks of the selected ages, and so interpolation is likely to have had only modest effects on the results. Only two other studies that we are aware of have frequent serial measures of growth between birth and 12 months[10,13], and our data were highly complete, enabling conditional regression analysis. Using the WHO growth standards, children of our study group had faster mean weight gain and faster mean linear growth between 1 and 5 years. However, we cannot say whether the associations that we found were due to the higher prevalence of faster growth in our population. We had detailed information about duration of exclusive breastfeeding and timing of introduction of complementary feeding.

A limitation was that, despite considerable effort, there was substantial loss to follow up (we were able to analyze 2227 children with complete growth data out of 5551 children in the original cohort). The analysis sample differed from the original cohort in several characteristics, which could bias our results if associations differed between those who did and did not take part in the study. Because length at birth is not always measured in the Netherlands we were not able to explore associations between birth length and childhood BP, and had to use birth weight as the anchor for our conditional measures. However, Adair et al.[14] compared results with and without the inclusion of birth length, and found that associations of subsequent relative weight gain and linear growth with later life BP were similar. Due to the observational nature of our study it is not possible to attribute causality or to exclude the possibility that the associations found are due to residual confounding. There is very little data on the relationship between childhood BP and later ‘hard’ disease outcomes like coronary heart disease and stroke. However, two studies have shown positive associations between adolescent blood pressure and either coronary artery disease[42] or coronary artery calcification (an indicator of atherosclerosis).[43] Finally, we studied a large number of associations, and some significant findings may have arisen by chance. This may be more of a concern with the infant feeding associations than with the growth associations, which were highly consistent.

## Conclusions

Faster growth after the age of one month and up to 5 years, in either weight or height is associated with higher childhood BP. While it would not seem advisable to limit infant linear growth, it seems reasonable to recommend avoiding consistent upward crossing of centiles for body weight in infancy and childhood, at least in high-income settings. There is still uncertainty about whether the association between breastfeeding and lower BP in later life is causal in nature. However, in light of other known health benefits[44], we think that promotion of a longer duration of breastfeeding is an important public health recommendation. Further studies are needed, particularly in populations in which influences on infant feeding practice and patterns of confounding factors differ, to assess the long-term effect of exclusive breastfeeding and timing and nature of the weaning diet.

## Supporting Information

S1 Dataset Dataset. (SAV)

S1 Table Weight and height z scores at the different time points using the WHO growth standards (www.who.int). (DOC)

S2 Table Relation of confounding variables with birth weight, relative weight gain and linear growth (in standard deviation scores). (DOCX)

S3 Table Relation of confounding variables with childhood blood pressure. (DOCX)

S4 Table Confounding variables by duration of breastfeeding. (DOCX)

S5 Table Confounding variables by timing of introduction of complementary feeding. (DOCX)

## Abbreviations

BP: blood pressure
YHC: Youth Health Care
