# Correction: Associations of Infant Feeding and Timing of Weight Gain and Linear Growth during Early Life with Childhood Blood Pressure: Findings from a Prospective Population Based Cohort Study

**DOI:** 10.1371/journal.pone.0168920

**Published:** 2016-12-19

**Authors:** Marieke de Beer, Tanja G. M. Vrijkotte, Caroline H. D. Fall, Manon van Eijsden, Clive Osmond, Reinoud J. B. J. Gemke

## Notice of Republication

This article was republished on December 5, 2016, as a Supporting Information file was published in error. The publisher apologizes for the error. Please download this article again to view the correct version.

## References

[1] de Beer M, Vrijkotte TGM, Fall CHD, van Eijsden M, Osmond C, Gemke RJBJ (2016) Associations of Infant Feeding and Timing of Weight Gain and Linear Growth during Early Life with Childhood Blood Pressure: Findings from a Prospective Population Based Cohort Study. PLoS ONE 11(11): e0166281 doi:10.1371/journal.pone.0166281 27832113 10.1371/journal.pone.0166281 PMC5104398

