# Supplementary material for: Associations of Infant Feeding and Timing of Weight Gain and Linear Growth during Early Life with Childhood Blood Pressure: Findings from a Prospective Population Based Cohort Study
Source: PLoS One. 2016 Nov 10;11(11):e0166281. doi: 10.1371/journal.pone.0166281 (PMC5104398; doi:10.1371/journal.pone.0166281)
Supplement: S1 Table — (DOC) [file pone.0166281.s002.doc]

**Supplemental material**

Associations of Infant Feeding and Timing of Weight Gain and Linear Growth During Early Life with Childhood Blood Pressure: Findings from a Prospective Population Based Cohort Study

**S1 Table. Weight and height z scores at the different time points using the WHO growth standards (www.who.int).**

|  | **Boys (n=1146)** | | **Girls (n=1081)** | |
| --- | --- | --- | --- | --- |
|  | Mean | Z score | Mean | Z score |
| **Child - measurements** |  |  |  |  |
| Weight (kg) |  |  |  |  |
| 0 months | 3.6 | 0.50 | 3.5 | 0.52 |
| 1 month | 4.6 | 0.21 | 4.3 | 0.19 |
| 3 months | 6.4 | 0.04 | 5.8 | -0.01 |
| 6 months | 8.1 | 0.17 | 7.5 | 0.17 |
| 12 months | 10.2 | 0.51 | 9.3 | 0.51 |
| 5 years | 21.2 | 1.03 | 21.0 | 0.94 |
| Height (cm) |  |  |  |  |
| 1 month | 55.2 | 0.28 | 54.1 | 0.24 |
| 3 months | 62.0 | 0.29 | 60.4 | 0.30 |
| 6 months | 68.4 | 0.35 | 66.6 | 0.37 |
| 12 months | 76.5 | 0.32 | 74.9 | 0.35 |
| 5 years | 116,9 | 1.50 | 116.2 | 1.43 |
