# Supplementary material for: Associations of Infant Feeding and Timing of Weight Gain and Linear Growth during Early Life with Childhood Blood Pressure: Findings from a Prospective Population Based Cohort Study
Source: PLoS One. 2016 Nov 10;11(11):e0166281. doi: 10.1371/journal.pone.0166281 (PMC5104398; doi:10.1371/journal.pone.0166281)
Supplement: S2 Table — (DOCX) [file pone.0166281.s003.docx]

**Supplemental material**

Associations of Infant Feeding and Timing of Weight Gain and Linear Growth During Early Life with Childhood Blood Pressure: Findings from a Prospective Population Based Cohort Study

**S2 Table. Relation of confounding variables with birth weight, relative weight gain and linear growth (in standard deviation scores).**

|  | **Birth weight** | |  | **Relative weight gain (z scores)** | | | | | | | | | | | | | | | |
| --- | --- | --- | --- | --- | --- | --- | --- | --- | --- | --- | --- | --- | --- | --- | --- | --- | --- | --- | --- |
|  |  |  |  | **0-1 m** | |  | **1-3 m** | |  | **3-6 m** | |  | **6-12 m** | |  | | **12 m-5 y** | | |
|  | B | P |  | B | P |  | B | P |  | B | P |  | B | P | |  | | B | P |
| **Mother** |  |  |  |  |  |  |  |  |  |  |  |  |  |  | |  | |  |  |
| Age (y) | 0.02 | <.001 |  | -0.02 | .001 |  | -0.02 | <.001 |  | -0.02 | .001 |  | 0.00 | .77 | |  | | -0.02 | .001 |
| BMI (kg/m^2^) | 0.03 | <.001 |  | 0.01 | .18 |  | 0.02 | <.001 |  | 0.01 | .03 |  | 0.00 | .95 | |  | | 0.05 | <.001 |
| Height (m) | 3.11 | <.001 |  | -0.33 | .28 |  | -0.55 | .07 |  | -0.18 | .56 |  | -0.57 | .06 | |  | | -1.96 | <.001 |
| Education (y) | 0.03 | <.001 |  | -0.01 | .08 |  | -0.03 | <.001 |  | -0.01 | .02 |  | 0.01 | .04 | |  | | -0.03 | <.001 |
| *Smoking* |  |  |  |  |  |  |  |  |  |  |  |  |  |  | |  | |  |  |
| No (ref) | - | - |  | - | - |  | - | - |  | - | - |  | - | - | |  | | - | - |
| 1-5 cigarettes/day | -0.33 | .006 |  | 0.22 | .06 |  | 0.20 | .09 |  | 0.20 | .09 |  | -0.09 | .47 | |  | | 0.14 | .23 |
| ≥ 6 cigarettes/day | -0.56 | <.001 |  | 0.04 | .78 |  | 0.28 | .04 |  | 0.33 | .01 |  | 0.06 | .69 | |  | | 0.26 | .06 |
| Alcohol, yes | 0.12 | .01 |  | -0.10 | .04 |  | -0.11 | .02 |  | 0.01 | .85 |  | 0.06 | .17 | |  | | -0.09 | .06 |
| Primiparity, yes | -0.27 | <.001 |  | -0.15 | <.001 |  | 0.08 | .06 |  | 0.05 | .24 |  | 0.01 | .85 | |  | | -0.01 | .80 |
| *Ethnicity* |  |  |  |  |  |  |  |  |  |  |  |  |  |  | |  | |  |  |
| Dutch (ref) | - | - |  | - | - |  | - | - |  | - | - |  | - | - | |  | | - | - |
| Surinamese | -0.46 | .001 |  | 0.08 | .55 |  | 0.29 | .03 |  | 0.07 | .62 |  | -0.41 | .003 | |  | | 0.26 | .06 |
| Turkish | -0.38 | .01 |  | 0.50 | .001 |  | 0.40 | .008 |  | 0.09 | .53 |  | 0.11 | .47 | |  | | 0.38 | .01 |
| Moroccan | -0.20 | .06 |  | 0.23 | .03 |  | 0.54 | <.001 |  | 0.47 | <.001 |  | 0.09 | .37 | |  | | 0.35 | .001 |
| Other | -0.22 | <.001 |  | 0.10 | .11 |  | 0.19 | .002 |  | -0.05 | .39 |  | -0.12 | .06 | |  | | 0.09 | .15 |
| *Hypertension* |  |  |  |  |  |  |  |  |  |  |  |  |  |  | |  | |  |  |
| None (ref) | - | - |  | - | - |  | - | - |  | - | - |  | - | - | |  | | - | - |
| Pre-existing | -0.11 | .43 |  | 0.04 | .78 |  | 0.37 | .008 |  | 0.03 | .86 |  | -0.14 | .32 | |  | | 0.07 | .60 |
| Gestational | -0.30 | <.001 |  | -0.08 | .27 |  | 0.09 | .23 |  | 0.06 | .41 |  | 0.03 | .67 | |  | | 0.18 | .02 |
| **Child - At birth** |  |  |  |  |  |  |  |  |  |  |  |  |  |  | |  | |  |  |
| Pregnancy duration (w) | 0.34 | <.001 |  | -0.01 | .61 |  | -0.11 | <.001 |  | 0.00 | .86 |  | -0.02 | .16 | |  | | 0.02 | .26 |

**S2 Table continued. Relation of confounding variables with birth weight, relative weight gain and linear growth (in standard deviation scores).**

|  | **Linear growth (z scores)** | | | | | | | | | | | | |
| --- | --- | --- | --- | --- | --- | --- | --- | --- | --- | --- | --- | --- | --- |
|  | **0-1 m** | |  | **1-3 m** | |  | **3-6 m** | |  | **6-12 m** | | **12 m-5 y** | |
|  | B | P |  | B | P |  | B | P |  | B | P | B | P |
| **Mother** |  |  |  |  |  |  |  |  |  |  |  |  |  |
| Age (y) | 0.00 | .36 |  | 0.00 | .88 |  | -0.02 | <.001 |  | -0.01 | .21 | 0.01 | .07 |
| BMI (kg/m^2^) | -0.02 | .003 |  | 0.00 | .49 |  | 0.01 | .11 |  | 0.01 | .22 | 0.01 | .02 |
| Height (m) | 2.67 | <.001 |  | 1.90 | <.001 |  | 1.02 | .001 |  | 1.30 | <.001 | 3.25 | <.001 |
| Education (y) | 0.02 | .005 |  | -0.01 | .11 |  | -0.03 | <.001 |  | -0.02 | .001 | 0.01 | .18 |
| *Smoking* |  |  |  |  |  |  |  |  |  |  |  |  |  |
| No (ref) | - | - |  | - | - |  | - | - |  | - | - | - | - |
| 1-5 cigarettes/day | -0.13 | .28 |  | 0.11 | .34 |  | 0.25 | .04 |  | 0.35 | .003 | -0.17 | .15 |
| ≥ 6 cigarettes/day | -0.54 | <.001 |  | -0.05 | .73 |  | 0.20 | .14 |  | 0.22 | .11 | 0.13 | .34 |
| Alcohol, yes | -0.03 | .56 |  | 0.00 | .94 |  | -0.12 | .01 |  | -0.07 | .15 | 0.01 | .83 |
| Primiparity, yes | 0.21 | <.001 |  | 0.15 | <.001 |  | 0.00 | 1.00 |  | 0.00 | .96 | 0.05 | .21 |
| *Ethnicity* |  |  |  |  |  |  |  |  |  |  |  |  |  |
| Dutch (ref) | - | - |  | - | - |  | - | - |  | - | - | - | - |
| Surinamese | -0.17 | .21 |  | -0.14 | .31 |  | 0.44 | .001 |  | 0.06 | .65 | 0.13 | .34 |
| Turkish | -0.06 | .69 |  | 0.06 | .68 |  | 0.34 | .02 |  | 0.02 | .88 | -0.64 | <.001 |
| Moroccan | -0.28 | .006 |  | -0.24 | .02 |  | 0.26 | .01 |  | 0.17 | .10 | -0.27 | .009 |
| Other | -0.07 | .24 |  | 0.07 | .24 |  | 0.06 | .36 |  | -0.02 | .71 | -0.18 | .005 |
| *Hypertension* |  |  |  |  |  |  |  |  |  |  |  |  |  |
| None (ref) | - | - |  | - | - |  | - | - |  | - | - | - | - |
| Pre-existing | 0.06 | .66 |  | -0.03 | .84 |  | -0.13 | .36 |  | 0.15 | .29 | 0.30 | .03 |
| Gestational | 0.05 | .50 |  | 0.01 | .92 |  | -0.03 | .70 |  | 0.09 | .22 | 0.04 | .64 |
| **Child - At birth** |  |  |  |  |  |  |  |  |  |  |  |  |  |
| Pregnancy duration (w) | 0.16 | <.001 |  | -0.01 | .48 |  | -0.07 | <.001 |  | -0.06 | .001 | -0.05 | 0.03 |

B values are linear regression coefficients indicating the relative weight gain and linear growth (in standard deviation scores) per unit change in the confounding variable (if a continuous variable) or between each category of the confounding variable and the reference category (if a categorical variable).
