# Supplementary material for: Associations of Infant Feeding and Timing of Weight Gain and Linear Growth during Early Life with Childhood Blood Pressure: Findings from a Prospective Population Based Cohort Study
Source: PLoS One. 2016 Nov 10;11(11):e0166281. doi: 10.1371/journal.pone.0166281 (PMC5104398; doi:10.1371/journal.pone.0166281)
Supplement: S3 Table — (DOCX) [file pone.0166281.s004.docx]

**Supplemental material**

Associations of Infant Feeding and Timing of Weight Gain and Linear Growth During Early Life with Childhood Blood Pressure: Findings from a Prospective Population Based Cohort Study

**S3 Table. Relation of confounding variables with childhood blood pressure.**

|  | **Systolic BP**  **(mm Hg)** | |  | **Diastolic BP**  **(mm Hg)** | |  |
| --- | --- | --- | --- | --- | --- | --- |
|  | B | P |  | B | P |  |
| **Mother** |  |  |  |  |  |  |
| Age (y) | -0.08 | .02 |  | -0.09 | .002 |  |
| BMI (kg/m^2^) | 0.21 | <.001 |  | 0.16 | <.001 |  |
| Height (m) | 3.78 | .10 |  | -1.94 | .31 |  |
| Education (y) | -0.15 | .002 |  | -0.15 | <.001 |  |
| Primiparous, yes | 0.16 | .60 |  | 0.29 | .24 |  |
| Alcohol, yes | -0.16 | .63 |  | -0.66 | .02 |  |
| *Smoking* |  |  |  |  |  |  |
| No (ref) | - | - |  | - | - |  |
| 1-5 cigarettes/day | 1.65 | .05 |  | 0.71 | .30 |  |
| ≥ 6 cigarettes/day | 0.93 | .32 |  | 1.10 | .13 |  |
| *Hypertension* |  |  |  |  |  |  |
| None (ref) | - | - |  | - | - |  |
| Pre-existing | 2.74 | .005 |  | 2.05 | .01 |  |
| Gestational | 0.92 | .08 |  | 0.82 | .06 |  |
| **Child - At birth** |  |  |  |  |  |  |
| Pregnancy duration (w) | -0.11 | .35 |  | -0.17 | .09 |  |

B values are linear regression coefficients indicating the change in BP (in mm Hg) per unit change in the confounding variable (if a continuous variable) or between each category of the confounding variable and its reference category (if a categorical variable) adjusting for the child’s age and sex and maternal ethnicity.

BP−blood pressure.
