# Supplementary material for: Associations of Infant Feeding and Timing of Weight Gain and Linear Growth during Early Life with Childhood Blood Pressure: Findings from a Prospective Population Based Cohort Study
Source: PLoS One. 2016 Nov 10;11(11):e0166281. doi: 10.1371/journal.pone.0166281 (PMC5104398; doi:10.1371/journal.pone.0166281)
Supplement: S4 Table — (DOCX) [file pone.0166281.s005.docx]

**Supplemental material**

Associations of Infant Feeding and Timing of Weight Gain and Linear Growth During Early Life with Childhood Blood Pressure: Findings from a Prospective Population Based Cohort Study

**S4 Table. Confounding variables by duration of exclusive breastfeeding.**

|  | **Duration of exclusive breastfeeding**  **(N=2217)** | | | | | | | |
| --- | --- | --- | --- | --- | --- | --- | --- | --- |
|  | **<1 m (reference)**  **(n=1185, 53.2%)** | | **1-3 m**  **(n=297, 13.3%)** | | **3-6 m**  **(n=429, 19.3%)** | | **>6 m**  **(n=316, 14.2%)** | |
| **Mother** | **(Mean, SD)** |  | **(Mean, SD)** | **B** | **(Mean, SD)** | **B** | **(Mean, SD)** | **B** |
| Age (y) | (32.0, 4.5) | - | (32.1, 4.3) | 0.14 | (32.5, 3.8) | 0.49 * | (32.7, 4.5) | 0.70 * |
| BMI (kg/m^2^) | (23.0, 3.8) | - | (22.7, 3.6) | -0.30 | (22.6, 3.3) | -0.42 * | (22.9, 3.7) | -0.11 |
| Height (m) | (1.70, 0.07) | - | (1.70, 0.07) | 0.00 | (1.70, 0.07) | 0.00 | (1.70, 0.07) | 0.01 |
| Education (y) | (9.7, 3.6) | - | (9.5, 3.5) | -0.21 | (10.8, 3.3) | 1.08 *** | (10.4, 3.7) | 0.69 ** |
| **Mother** | **Col %** |  | **Col %** | **OR** | **Col %** | **OR** | **Col %** | **OR** |
| Primiparous, yes | 56.6 | - | 54.9 | 0.93 | 56.4 | 0.99 | 46.2 | 0.66 ** |
| Alcohol, yes | 28.9 | - | 29.3 | 1.02 | 30.4 | 1.08 | 22.2 | 0.70 * |
| *Smoking* |  |  |  |  |  |  |  |  |
| No (reference) | 92.2 | - | 94.3 | - | 97.2 | - | 97.5 | - |
| 1-5 cigarettes/day | 4.4 | - | 3.4 | 0.75 | 1.9 | 0.40 * | 1.3 | 0.27 * |
| ≥ 6 cigarettes/day | 3.5 | - | 2.4 | 0.67 | 0.9 | 0.26 ** | 1.3 | 0.35 * |
| *Hypertension* |  |  |  |  |  |  |  |  |
| None (reference) | 88.4 | - | 90.8 | - | 90.9 | - | 87.3 | - |
| Pre-existing | 2.9 | - | 3.1 | 1.03 | 0.9 | 0.32 * | 1.9 | 0.67 |
| Gestational | 8.7 | - | 6.1 | 0.68 | 8.2 | 0.91 | 10.8 | 1.25 |
| **Mother** | **Row %** |  | **Row %** | **OR** | **Row %** | **OR** | **Row %** | **OR** |
| *Ethnicity* |  |  |  |  |  |  |  |  |
| Dutch (reference) | 52.9 | - | 13.7 | - | 19.9 | - | 13.5 | - |
| Surinamese | 68.5 | - | 16.7 | 0.94 | 5.6 | 0.22 * | 9.3 | 0.53 |
| Turkish | 55.6 | - | 17.8 | 1.24 | 20.0 | 0.96 | 6.7 | 0.47 |
| Moroccan | 62.2 | - | 10.2 | 0.64 | 9.2 | 0.39 * | 18.4 | 1.16 |
| Other | 48.6 | - | 11.1 | 0.89 | 21.3 | 1.16 | 18.9 | 1.53 * |
| **Child - At birth** | **(Mean, SD)** |  | **(Mean, SD)** | **B** | **(Mean, SD)** | **B** | **(Mean, SD)** | **B** |
| Pregnancy duration (w) | (40.1, 1.2) | - | (40.1, 1.2) | 0.03 | (40.3, 1.2) | 0.22 ** | (40.2, 1.3) | 0.10 |
| Birth weight (kg) | (3.53, 0.50) | - | (3.53, 0.46) | 0.00 | (3.57, 0.46) | 0.04 | (3.58, 0.48) | 0.04 |

B values are linear regression coefficients indicating the change in the confounding variable (if a continuous variable) for each category of the breastfeeding variable compared with the reference category. OR is the corresponding odds ratio (if a categorical variable). *P<0.05, **P<0.01, ***P<0.001.
