# Supplementary material for: Associations of Infant Feeding and Timing of Weight Gain and Linear Growth during Early Life with Childhood Blood Pressure: Findings from a Prospective Population Based Cohort Study
Source: PLoS One. 2016 Nov 10;11(11):e0166281. doi: 10.1371/journal.pone.0166281 (PMC5104398; doi:10.1371/journal.pone.0166281)
Supplement: S5 Table — (DOCX) [file pone.0166281.s006.docx]

**Supplemental material**

Associations of Infant Feeding and Timing of Weight Gain and Linear Growth During Early Life with Childhood Blood Pressure: Findings from a Prospective Population Based Cohort Study

**S5 Table. Confounding variables by timing of introduction of complementary feeding.**

|  | **Timing of introduction of complementary feeding**  **(N=2203)** | | | | | |
| --- | --- | --- | --- | --- | --- | --- |
|  | **<4 m**  **(n=117, 5.3%)** | | **4-6 m**  **(n=826, 37.5%)** | | **>6 m (reference)**  **(n=1260, 57.2%)** | |
| **Mother** | **(Mean, SD)** | **B** | **(Mean, SD)** | **B** | **(Mean, SD)** |  |
| Age (y) | (32.2, 5.1) | -0.21 | (31.9, 4.4) | -0.48 * | (32.4, 4.3) | - |
| BMI (kg/m^2^) | (23.6, 4.0) | 0.87 * | (23.0, 3.8) | 0.24 | (22.7, 3.5) | - |
| Height (m) | (1.69, 0.07) | -0.01 | (1.70, 0.07) | -0.00 | (1.70, 0.07) | - |
| Education (y) | (8.7, 3.5) | -1.43 *** | (9.9, 3.6) | -0.27 | (10.1, 3.6) | - |
| **Mother** | **Col %** | **OR** | **Col %** | **OR** | **Col %** |  |
| Primiparous, yes | 47.9 | 0.76 | 55.7 | 1.04 | 54.8 | - |
| Alcohol, yes | 25.6 | 0.93 | 29.8 | 1.14 | 27.2 | - |
| *Smoking* |  |  |  |  |  |  |
| No (reference) | 86.3 | - | 93.1 | - | 95.6 | - |
| 1-5 cigarettes/day | 7.7 | 3.25 ** | 3.8 | 1.47 | 2.6 | - |
| ≥ 6 cigarettes/day | 6.0 | 3.63 ** | 3.1 | 1.77 * | 1.8 | - |
| *Hypertension* |  |  |  |  |  |  |
| None (reference) | 84.5 | - | 90.0 | - | 88.9 | - |
| Pre-existing | 6.0 | 2.95 * | 2.2 | 1.00 | 2.1 | - |
| Gestational | 9.5 | 1.11 | 7.8 | 0.85 | 9.0 | - |
| **Mother** | **Row %** | **OR** | **Row %** | **OR** | **Row %** |  |
| *Ethnicity* |  |  |  |  |  |  |
| Dutch (reference) | 5.3 | - | 37.3 | - | 57.3 | - |
| Surinamese | 14.8 | 3.45 ** | 38.9 | 1.29 | 46.3 | - |
| Turkish | 4.4 | 0.90 | 42.2 | 1.22 | 53.3 | - |
| Moroccan | 7.1 | 1.57 | 43.9 | 1.38 | 49.0 | - |
| Other | 3.1 | 0.53 | 35.3 | 0.88 | 61.7 | - |
| **Child - At birth** | **(Mean, SD)** | **B** | **(Mean, SD)** | **B** | **(Mean, SD)** |  |
| Pregnancy duration (w) | (39.9, 1.2) | -0.22 | (40.1, 1.2) | -0.04 | (40.1, 1.2) | - |
| Birth weight (kg) | (3.49, 0.51) | -0.06 | (3.56, 0.49) | 0.02 | (3.54, 0.48) | - |

B values are linear regression coefficients indicating the change in the confounding variable (if a continuous variable) for each category of the complementary feeding variable compared with the reference category. OR is the corresponding odds ratio (if a categorical variable). *P<0.05, **P<0.01, ***P<0.001.
